# Treatment of Loss of Sexual Power by Ligation of Veins

**Published:** 1893-10

**Authors:** 


### TREATMENT OF LOSS OF SEXUAL POWER BY LIGATION OF VEINS.

---

The loss of sexual powers, says Dr. Alfred King, (*Boston Medical and Surgical Journal,*) or rather deficient erections of the penis, render so many men miserable mentally and physically, that any new method of treatment, promising a radical cure, merits investigation and trial:

Three immediate causes of deficient erections may be specified: destruction of the erector muscles, loss of nerve power, and a change in the circulation. The first of these is so rare and so easily determined that it needs only a passing notice. The second cause, loss of nerve power, seems to me to have received more prominence than it deserves, as it is the basis upon which all treatment is founded. While its force in many cases is undisputed, yet the frequent failure of treatment based upon it leads me to direct attention to the importance of the third cause, that is, a change in the circulation. This change takes place in the veins, especially those which do not pass beneath the pubic arch, or are not acted upon by the erector muscles. Repeated engorgement of the penis renders their caliber larger, and, consequently, there is a more rapid escape of blood through them. When therefore, an erection takes place, it cannot be maintained on account of the escape of blood through these channels. Thus we have the history of the gradual shortening of the duration of erections, and finally, scarcely none, if any, as these veins grow larger.

The remedy for such a condition, especially when far advanced, is not in the use of drugs, but may be brought about speedily and safely by the ligation of some of the larger of these veins.

The following case is given to illustrate this cause and its successful treatment:

Mr. M., aged thirty-five, a laborer of powerful physique, came to me about a year ago with the following history: For several years he had been losing the power of maintaining an erection; during the past year its duration had becn so short that sexual intercourse had been rendered impossible. There was a loss of sexual desire and great mental depression. Excessive use or abuse was the cause of this condition.

I gave all possible encouragement to the patient; advised total abstinence from sexual intercourse, cold baths, (especially to the spine and external genitals;) prescribed bromides, canabis indica, cantharides, damiana, phosphorus and salts containing it; pushed strychnine as far as it could be borne; gave various tonics; used electricity; and in short did everything which offered any hope of success, but all to no effect so far as producing any stronger erections was concerned.

Careful study of the case convinced me that the immediate cause of the trouble was a physical one, due to a leakage, as it were, or to a too rapid escape of blood from the penis when erected. I therefore determined to ligate a couple of the larger subcutaneous veins at the base of the penis and watch the effect.

This was very easily done by the use of cocaine. A vein on each side of the penis was exposed, ligated in two places and severed between the ligatures. A dressing was lightly applied and held in position by a strip of adhesive plaster placed longitudinally. The result was immediate. In less than five minutes after leaving my office he had an erection. That night he was awakened by a powerful erection which made the bandage so painfully tight that he was obliged to jump out of bed onto the cold floor to subdue it. Primary union was prevented by the frequent erections, but the success of the operation was certain.

Two months later he reported himself well, mentally and physically, his sexual appetite had returned and since the operation his power of maintaining erections had been as good as ever.—*Med. and Surg. Reporter.*

